# Highly efficient stacking ensemble learning model for automated keratoconus screening

**DOI:** 10.1186/s40662-025-00440-6

**Published:** 2025-06-24

**Authors:** Zahra J. Muhsin, Rami Qahwaji, Ibrahim Ghafir, Mo’ath AlShawabkeh, Muawyah Al Bdour, Saif Aldeen AlRyalat, Majid Al-Taee

**Affiliations:** 1 Faculty of Engineering and Digital Technologies, University of Bradford, Bradford, BD7 1DP UK; 2 Department of Ophthalmology, The Hashemite University, Zarqa, Jordan; 3 School of Medicine, The University of Jordan, Amman, Jordan; 4 Independent Consultant of Computing and Systems Engineering, Liverpool, UK

**Keywords:** Corneal tomography, Ensemble learning, Feature selection, Keratoconus screening, Stacking ensemble learning

## Abstract

**Background:**

Despite extensive research on keratoconus (KC) detection with traditional machine learning models, stacking ensemble learning approaches remain underexplored. This paper presents a stacking ensemble learning method to enhance automated KC screening.

**Methods:**

This study utilizes a clinical dataset containing detailed corneal data from 2491 cases classified as non-KC (NKC), subclinical KC (SCKC) and clinical KC (CKC). Each cornea is represented by 79 features extracted from Pentacam imaging. Following extensive pre-processing, key corneal features that are strongly correlated with the target diagnosis are identified. These features are the keratometry of the steepest anterior point, surface variance index, vertical asymmetry index, height decentration index, and height asymmetry index. A novel stacking ensemble model is developed using the selected features to improve corneal classification into NKC, SCKC, and CKC by integrating top tree-based classifiers (random forest, gradient boosting, decision trees) with a support vector machine meta-classifier.

**Results:**

The pre-processing and feature selection techniques reduced the model's parameters to just 6.33% of the original dataset, improving classification performance, and cutting over 85% of the training time. The performance of the developed model was validated and tested on unseen data. Experimental results showed that the model outperforms existing studies, achieving 99.72% accuracy, precision, sensitivity, F1, and F2 scores, with a Matthews correlation coefficient of 0.995. It accurately classified all NKC and CKC cases, with just one misclassification involving an SCKC case. The model also demonstrated consistent performance on 100 additional unseen test cases, underscoring its generalizability and robustness in KC screening.

**Conclusions:**

By combining the strengths of diverse base models and key Pentacam indices, the stacking ensemble approach ensures reliable, accurate KC screening, providing clinicians with an automated tool for early detection and better patient management.

**Supplementary Information:**

The online version contains supplementary material available at 10.1186/s40662-025-00440-6.

## Background

Keratoconus (KC) is a prevalent eye disorder characterized by the progressive thinning of the cornea, changing its shape from dome to cone-like. This can lead to impaired vision, astigmatism, and a resultant diminished quality of life [1, 2]. Both sexes are affected, with the condition typically appearing in early adolescence and progressing until around the fourth decade of life. Despite decades of research, the exact cause of keratoconus remains unclear. However, it is believed that a combination of environmental and genetic factors contributes to the development and progression of KC.

The incidence and prevalence of KC vary significantly due to selection biases, differences in study populations and diagnostic criteria, and disparities in access to ophthalmic care [3, 4], making cross-study comparisons challenging. However, there is evidence to suggest that the incidence of KC is higher among Middle Eastern and South Asian communities when compared with other groups [5, 6]. Reported studies suggest that KC prevalence ranges from 0.0002% in Russia’s Urals to 4.79% among Saudi adolescents in Riyadh [4]. These findings suggest that specific populations require resource allocation and increased attention to future screening programs. Additionally, most epidemiologic studies on KC have focused on patients in clinic or hospital settings, where data collection is relatively straightforward. This approach likely underestimates the true prevalence of the disease as many patients are asymptomatic which leads to early and subtle manifestations being overlooked [7, 8]. To accurately determine the true prevalence of KC, population-based screening is essential for capturing its full spectrum.

Recent advancements in corneal topography and the integration of machine learning, along with increased awareness, have the potential to facilitate earlier detection of KC, especially in its subclinical stage [9]. Early diagnosis is crucial for managing symptoms of reduced visual acuity and astigmatism, as well as for preventing further disease progression [10]. However, detecting KC in its early stages remains challenging due to the absence of symptoms, with eyeglasses or contact lenses often providing sufficient visual correction. Moreover, treatment strategies for KC vary based on the disease stage and may involve both non-surgical and surgical interventions [11].

KC diagnosis relies on medical history, corneal imaging, and physical examination, including refractive assessment, retinoscopy, and slit-lamp exam [12]. The commonly used devices for corneal imaging include corneal topography, corneal tomography, and optical coherence tomography [13]. Together, these imaging techniques are essential in diagnosing and monitoring corneal diseases like KC, offering detailed insights into corneal health and integrity. However, during the early stages, slit-lamp exam is typically non-KC (NKC) and therefore is unable to show any suspicious signs [14]. Moreover, visual acuity and refraction are also not significantly affected during the early stages. As a result, accurate screening and diagnosis require the combined use of multiple devices [15]. If KC is suspected, clinicians are encouraged to employ additional diagnostic methods, even if one technique shows no clear abnormalities. Using multiple approaches together or independently helps identify KC in its early stages [16, 17].

Recent advancements in machine learning (ML) [18, 19] have become valuable tools for identifying and diagnosing complex diseases, including KC early detection and severity staging [3, 20]. A range of ML methods have been proposed specifically for KC diagnosis [18]. Supervised methods use labeled input data to detect KC from unlabeled data [21], while unsupervised methods identify underlying patterns or clusters within datasets [22]. Deep learning including convolutional neural networks (CNNs) architectures, a subset of ML [23], has also shown potential for early KC detection using corneal topographic maps [24, 25]. CNNs are specialized for processing grid-like data such as images, using convolutional layers to automatically extract hierarchical features like edges, textures, and objects, minimizing manual feature engineering. However, their performance heavily depends on the availability of large datasets for training [17], which remains a challenge in ML-based KC studies.

These ML methods detect KC by analyzing various types of data obtained from corneal imaging devices along with other clinical and biometric variables [26]. By utilizing corneal topography, tomographic data, or a combination of both, these techniques can effectively distinguish between different classes of corneal abnormalities [27]. In the context of KC screening, which differentiates between NKC, subclinical KC (SCKC), and clinical KC (CKC) conditions, various machine learning (ML) models have been developed using different corneal imaging devices, datasets and input features. Issarti et al. [28] proposed a feedforward neural network (FNN), trained on a local dataset of 851 subjects with five selected features. It achieved 96.56% accuracy, with a sensitivity of 97.78% and specificity of 95.56%. Similarly, Shi et al. [29] employed an artificial neural network (ANN) alongside other models, utilizing a local dataset of 121 subjects and 49 selected features. While accuracy was not reported, the study achieved a sensitivity of 98.5%, a specificity of 94.7%, and an area under the receiver operating characteristic curve (AUC) of 93%.

Both Lavric et al. [30] and Shanthi et al. [31] implemented support vector machine (SVM) models, using a public dataset of 3,151 subjects and a local dataset of 205 subjects, respectively. Lavric et al.'s model with eight input features reached 94% accuracy, with 87% sensitivity and 98% specificity, while Shanthi et al.'s model with five input features achieved 91.8% accuracy, with high sensitivity (94.2%) and specificity (97.5%). Malyugin et al. [32] introduced a quadratic discriminant analysis (QDA) model using a large local dataset of 47,419 subjects and seven input features. The model demonstrated a classification capability with an AUC of 95%, though specific accuracy and sensitivity values were not reported. Song et al. [33] proposed a decision tree (DT) model trained on a local dataset of 194 subjects and 20 input features, achieving 92.4% accuracy, with a sensitivity of 90.3% and specificity of 94.3%.

Among ML models for KC detection, random forest (RF) has been the most widely used, as reported by Cao et al. [34], Aatil et al. [35], Herber et al. [36], Castro-Luna et al. [37], Cao et al. [38], and Muhsin et al. [39]. These studies utilized varying dataset sizes, input features, and achieved different levels of classification performance. Notably, the most recent study by Muhsin et al. [32] reported the highest accuracy of 99.6%, with five input features, a sensitivity of 99.01% and a precision of 99.72%. Although individual ML methods have shown significant promise in detecting KC [40], there is still a notable gap in the exploration of more advanced techniques, such as stacking ensemble learning.

Stacking ensemble learning integrates predictions from multiple models to produce a robust and more accurate outcome [41–43], offering significant potential for enhancing diagnostic performance by leveraging the complementary strengths of various algorithms [44]. This approach has recently been proposed and implemented in various ophthalmology applications including refraction prediction in cataract surgery [45], cataract grading [46], clinical fitting of orthokeratology lens for myopia correction [47], and classification of glaucoma and diabetic retinopathy [48]. However, the effectiveness of this learning approach has not yet been explored in KC diagnosis. Further research into advanced ensemble learning techniques for KC diagnosis could therefore improve KC detection, especially in its early stages, where subtle variations in corneal structure may be missed by traditional ML methods.

In contrast to earlier studies on KC that primarily concentrated on individual base ML models, this work explores the potential of a stacking ensemble learning approach for KC screening. The proposed screening tool, developed in collaboration between ML experts and ophthalmologists, represents a significant advancement by integrating multiple models to enhance diagnostic accuracy. This builds on the findings of a recent study by the authors [39], which investigated and compared the performance of individual base learning models in distinguishing between NKC, SCKC, and CKC corneas. By shifting the focus from single-model approaches to a more advanced ensemble method, this study aims to provide a more robust and accurate screening tool. The close collaboration between clinical and data science experts ensures that the tool is not only technically sound but also aligned with practical diagnostic needs in ophthalmology.

The main contributions of this study are: (i) presenting a comprehensive approach for collecting and pre-processing of a raw clinical dataset, (ii) identifying and selecting the most relevant corneal tomography features that contribute significantly to the classification performance, (iii) developing a novel stacking ensemble model using top-performing base models and a carefully chosen subset of key corneal tomography indices, and (iv) providing a performance comparison between the proposed model and state-of-the-art results.

## Methods

**Fig. 1:**
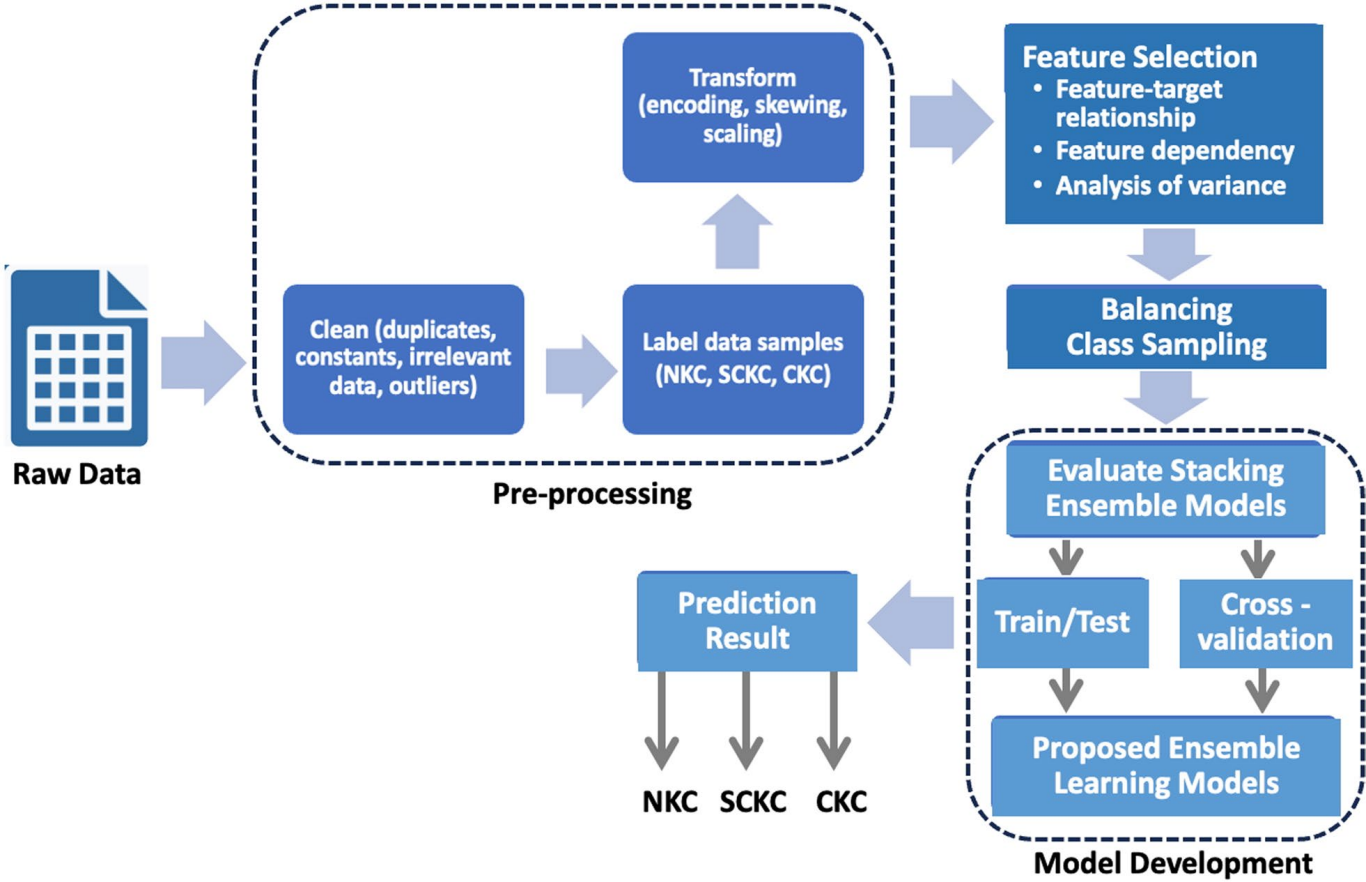
Workflow of the development methodology

This section presents an overview of the proposed development methodology and study dataset, followed by a detailed breakdown of each phase, including preprocessing, feature selection, class balancing, and the modeling and validation stages for a stacking ensemble learning model. Figure 1 illustrates the workflow of this methodology. The process was carried out collaboratively between ML experts and ophthalmologists in an iterative manner [49], ensuring clinical validity and alignment with standard practices.

### Development methodology

The development process begins with the manual collection of the study dataset from the Pentacam imaging devices [50, 51], a diagnostic device that provides detailed corneal imaging data. Once the dataset is gathered, it undergoes a pre-processing stage to ensure the quality and accuracy of the input. Following this, a feature selection phase is conducted, where the most informative and diagnostically significant features were selected, reducing the dimensionality of the dataset and improving model efficiency. This step ensures that the most relevant features to the target diagnosis are retained, resulting in a more focused and robust dataset. With the refined subset of corneal features, various combinations of base learners are then trained and validated toward developing a combination of stacking ensemble learning models. Finally, the best-performing stacking model, determined through extensive performance evaluation, is selected for further analysis. The performance of the proposed approach is then evaluated, and the results are discussed, highlighting the model's effectiveness in KC screening using the selected features.

### Dataset description

The dataset was collected over a ten-year period, from 2013 to 2023, at two Jordanian eye-care facilities: Jordan University Hospital and Al-Taif Eye Center. Ethics approval was obtained from the ethics committees of both facilities, with protocols JUH-2023-1593/67 for Jordan University Hospital and ATEC-GM/15 for Al-Taif Eye Center. The dataset includes detailed data from 2,491 corneas, categorized into three groups: NKC, SCKC, and CKC. An additional 100 corneas, distributed across different categories, were reserved as unseen data to evaluate the proposed model. Each cornea in the dataset is associated with 79 feature columns, capturing a wide range of variables derived from Pentacam corneal imaging devices.

The data collection process involved several key steps to ensure data integrity and confidentiality:

- *Acquisition of corneal data*—corneal data were obtained using Pentacam devices, which provide detailed topographic and tomographic measurements of the cornea.
- *Missing data*—incomplete data resulting from collection errors, clerical errors, or device malfunctions, were removed by the medical team before pre-processing.
- *Linkage to patient records*—the collected data were meticulously linked to corresponding patient records to ensure accurate correlation with diagnostic outcomes.
- *Exclusion of personal follow-up data*—any personal follow-up information that could identify patients was excluded to protect privacy and maintain confidentiality.
- *Anonymization and secure storage*—the data were anonymized by healthcare professionals to prevent patient identification. Subsequently, the anonymized data were stored in a Microsoft Excel file and encrypted, ensuring both data integrity and privacy.

### Pre-processing

The initial pre-processing of the study dataset was carried out by the clinicians involved in its collection as mentioned in the data collection process. This section further refined the data to enhance its quality. The additional pre-processing steps included data cleaning, labeling, and transformation techniques, as outlined below.

#### Data cleaning

Several data cleaning techniques were applied to the dataset to prepare it for subsequent analysis and ML modeling. These techniques are outlined as follows.

- *Eliminating duplication*: Redundant data elements, including those with identical values or derived from other parameters, are removed in consultation with ophthalmologists. This crucial step in reducing the number of dataset features is detailed later in the feature selection section.
- *Excluding constant data elements*: Constant features devoid of informative variation, are omitted as they offer no value to the ML model. Approximately 5% of the dataset’s features, attributed to imaging device errors, were removed due to their constant values.
- *Filtering irrelevant parameters*: Medically irrelevant features, such as examination time or clinical comments, are removed from the dataset. The filtered features represent 21.5% of the dataset’s features.
- *Removing outliers*: Observations significantly distant from others are excluded, identifiable through visual inspection or statistical calculations. This technique is applied to all measurements of the remaining features. Further details on the removal of outliers are reported in [52].

Following the implementation of these pre-processing steps, the dataset's feature columns were reduced from 79 to 51.

#### Feature transformation

This stage involved the employment of several feature transformation techniques: encoding, skewing, and scaling.

*Feature encoding*: Involves converting non-numeric values into numeric ones, a crucial step for handling categorical features that represent qualitative data without inherent mathematical significance. While such data is easily interpreted by humans, it poses challenges for computational models, which require numeric input to perform calculations and analysis. Consequently, all categorical data were converted into numerical formats. Nominal features were encoded using binary or one-hot encoding (0, 1), while ordinal features used ordinal encoding (1, 2, ... n). For instance, numerical values (0, 1, 2) replaced the diagnosis labels NKC, SCKC, and CKC, respectively.

*Skew transformation*: Raw datasets often exhibit skewness, indicating they can be positively skewed (peaking to the right) or negatively skewed (peaking to the left), thereby deviating from a normal distribution. Many statistical tests, such as analysis of variance and the F-test, assume that the data approximates a normal distribution. However, the current dataset demonstrates significant asymmetry, with values falling well outside the typical acceptable range for normality, which is between +2 and −2 [53]. Addressing this skewness is crucial to ensure that the dataset meets the assumptions of these statistical tests and to improve the reliability of subsequent analyses. Various transformation techniques were applied to correct the skewness, including log transformation, Box-Cox transformation, and square root (SQRT) transformation. Among these, the SQRT transformation proved to be the most effective, successfully bringing the skewed features within the acceptable range for normality. This adjustment enhances the dataset, thereby improving the validity and robustness of statistical analyses and M models applied to the data.

*Feature scaling*: In statistical feature selection methods, such as analysis of variance (ANOVA), feature scaling is necessary because features with larger magnitudes may dominate the F-statistic, leading to biased selection [54]. Additionally, scaling is essential for non-tree-based algorithms like logistic regression (LoR), SVMs, K-nearest neighbors (KNNs), which rely on distance calculations or assumptions about data distribution. Without scaling, features on different scales can distort results, causing inaccuracies or skewed interpretations in these models. As the proposed ensemble model combines both types, scaling ensures numerical stability and balanced feature contributions for optimal performance. In this study, the standard scaler is employed due to its ability to normalize both positive and negative feature values, which is the case in the study dataset.

#### Data labeling

A team of three ophthalmologists meticulously labeled the elements of the collected dataset based on the guidelines established in the 2015 Global Consensus on KC diagnosis [11]. This labeling procedure involved a comprehensive evaluation process that included clinical, optometric, and ophthalmic examinations. These examinations encompassed a range of diagnostic tools and techniques, such as slit-lamp microscopy, retinoscopy, and corneal tomography. The ophthalmologists involved in the labeling process demonstrated a high level of consistency, achieving an agreement rate exceeding 97% in their classifications. Their classification labels include three distinct categories: NKC (1836 samples); SCKC (171 samples), and CKC corneas (484 samples). Table 1 provides concise definitions of these conditions; each paired with a representative sagittal curvature image (front view) corresponding to the respective diagnosis.

**Table 1:** Clinical characteristics of the diagnostic labels

Representative image	Description
	Non-keratoconus (NKC) cornea—in NKC cases, there is an absence of any tomographic abnormalities on corneal imaging. Corneal curvature, elevation, and pachymetry maps appear within standard parameters, indicating a structurally healthy and uniformly shaped cornea without signs of thinning, steepening, or irregular elevation. The best-corrected visual acuity (BCVA) in these cases is 20/20 or better. Clinically, no signs of KC are detected during the optometric assessment, where both subjective and objective refractions confirm the absence of irregular astigmatism. Retinoscopy shows a normal light reflex without the characteristic scissoring reflex seen in keratoconus. Slit-lamp examination reveals a clear and smooth corneal surface, without any signs of distortion, thinning, striae, or scarring. These findings differentiate NKC cornea from subclinical keratoconus (SCKC) or clinical keratoconus (CKC) corneas [51, 55]
	SCKC cornea—in SCKC, subtle tomographic abnormalities include key findings such as increased posterior corneal elevation, which reflects early structural changes that may not yet manifest in clinical symptoms. Localized thinning in the corneal pachymetry map is another common indicator, suggesting areas of focal weakening, while steepening of the anterior corneal surface highlights the beginning stages of corneal deformation. Additionally, there is often asymmetry between the anterior and posterior corneal surfaces, which signifies early distortion in the cornea's shape and structure. Despite these tomographic irregularities, the BCVA remains 20/20 or better, with no or minimal signs of KC on standard examinations, including optometric testing, retinoscopy evaluation and slit-lamp exam [51, 56]
	CKC cornea**—**in CKC, frank tomographic abnormalities are evident across various corneal maps, including curvature, elevation, and pachymetry maps. On curvature maps, there is marked steepening, often centrally or infero-temporally, which reflects significant deformation of the corneal shape. Elevation maps typically reveal abnormal rises in both anterior and posterior surfaces, particularly in posterior elevation, while pachymetry maps display areas of localized thinning, most commonly at or near the cone apex, highlighting regions of structural weakness in the cornea. These findings correlate with decreased BCVA due to the irregular corneal shape. Irregular astigmatism is common, and retinoscopy often shows a scissoring reflex. Additionally, the slit-lamp exam further confirms the diagnosis by identifying corneal features typical of CKC, such as corneal striae or scar [51, 57]

### Feature selection

The feature selection process involves an analysis of feature-target relationships, feature dependencies, and variance. This approach combines statistical methods with the expertise of ophthalmologists to ensure a comprehensive and informed selection of features.

#### Feature-target relationship

To examine feature-target relationships, Pearson’s method [58] was used to calculate the correlation coefficients of the dataset features. These coefficients, ranging from –1 to 1, indicate the direction and strength of the relationship between each feature and the target diagnosis. Based on this analysis, several features demonstrated weak or no correlation with the target. Features with correlation coefficients between –0.5 and 0.5 were deemed less important and were safely removed, leading to a reduction in the feature columns from 51 to 39.

#### Feature dependency

Some corneal features that either directly or indirectly rely on primary features were identified and validated with the support of expert ophthalmologists. This collaborative approach ensured that the features were not only relevant but also clinically significant. Examples of these features include [59]:

- Minimum sagittal curvature (RminSag): This feature is dependent on the minimum radius of curvature on the front surface (Rmin (mm)).
- Minimum corneal radius values, both front (Rmin (mm)) and back (Rmin_B (mm)) surfaces: These features are influenced by the keratometry measurement of the steepest point on the anterior surface (Kmax (D)).
- Radius of the cornea's back surface (Rs_B (mm)) and the second steepest keratometry at the back (K2_B (D)): These measurements are dependent upon one another.
- Steep keratometry at the front (K2_F (D)) and the sagittal radius of curvature of the anterior corneal surface (Rs_F (mm)): These features are related as products of each other.
- Posterior corneal curvature (Km_B (D)) and flat keratometry (back) (K1_B (D)): These measurements are dependent on the flat radius of curvature at the back (Rf_B (mm)).
- Flat keratometry at the front (K1_F (D)) and anterior corneal surface curvature (Km_F (D)): These features depend on the flattest radius of anterior corneal curvature (Rf_F (mm)).

After carefully analyzing these dependencies and other related factors, the initial set of 39 features was refined and reduced to 32 features.

#### Analysis of variance

Numerous methods for feature selection exist, typically categorized as filter-based, wrapper, and embedded techniques [60, 61]. Among these, filter-based techniques are particularly appealing due to their independence from classifiers, computational efficiency, scalability to datasets with numerous characteristics, and others [62]. In this study, a filter method utilizing analysis of variance (ANOVA) [54] is employed to examine the connection between diagnosis and each feature. ANOVA is a statistical technique that compares the means of multiple subgroups to assess potential similarities or differences in specific aspects across the study samples.

**Fig. 2:**
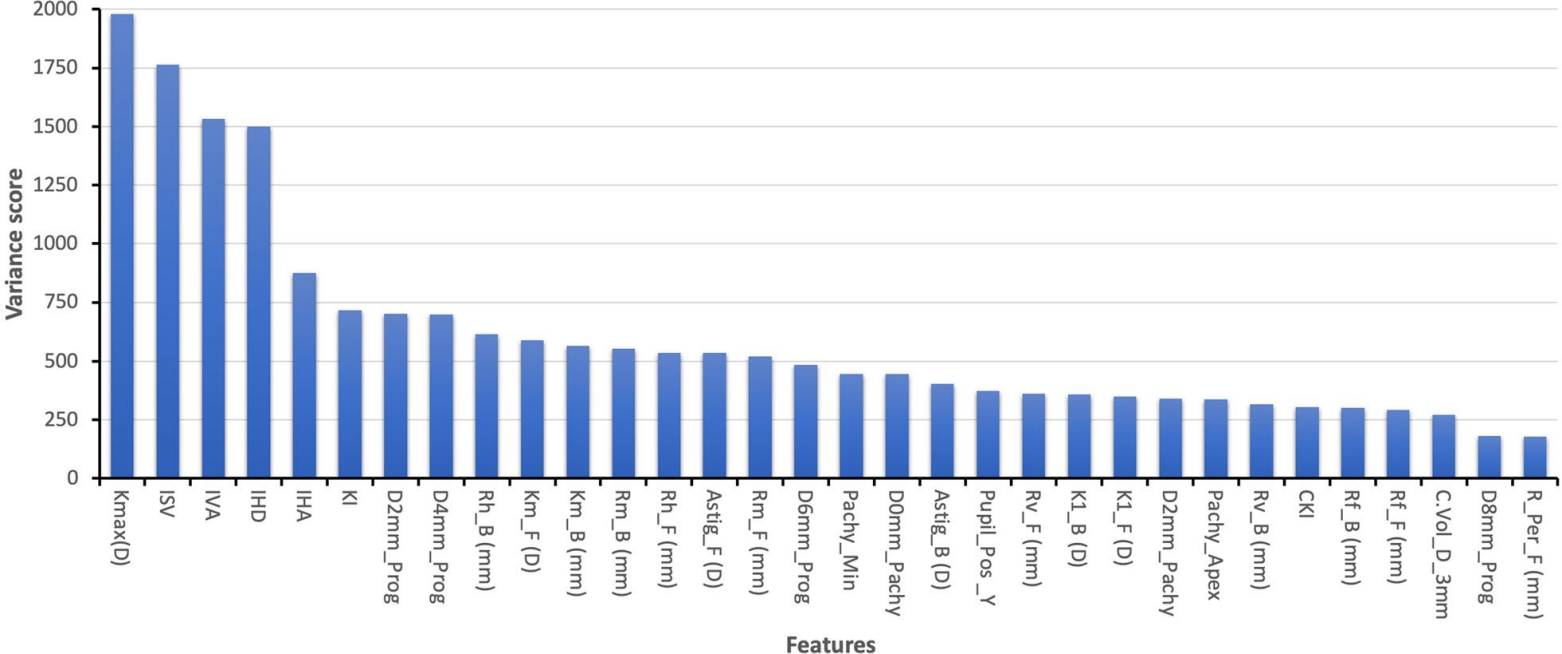
Variance scores of the derived feature set (n = 32). Kmax (D), keratometry of the steepest point (anterior); ISV, index of surface variance; IVA, index of vertical asymmetry; IHD index of height decentration; IHA, index of height asymmetry; KI, keratoconus index; D2mm_Prog, pachymetric progression in a 2-mm diameter zone around the cornea's thinnest point; D4mm_Prog, pachymetric progression in a 4-mm diameter zone around the cornea's thinnest point; Rh_B (mm), horizontal radius of curvature of the cornea (back); Km_F (D), anterior corneal surface; Km_B (mm), posterior corneal curvature; Rm_B (mm), curvature radius of the back surface of the cornea (posterior); Rh_F (mm), central radius in horizontal direction (anterior); Astig_F (D), central corneal astigmatism (anterior corneal values measured in diopters); Rh_F (mm), central radius in horizontal direction (anterior); Rm_F (mm), curvature radius of the front surface of the cornea (anterior); D6mm_Prog, pachymetric progression in a 6-mm diameter zone around the cornea's thinnest point; Patchy_Min, thinnest pachymetry (μm); D0mm_Patchy, average pachymetry on concentric rings with radii 0 (mm); Astig_B (D), central corneal astigmatism (posterior corneal values measured in diopters); Pupil_Pos_Y, y‐coordinates of the pupil position relative to the corneal apex; Rv_F (mm), central radius in vertical direction (anterior); K1_B (D), flat keratometry (back) measured in dioptres; K1_F (D), flat keratometry (front) measured in dioptres; D2mm_Patchy, average pachymetry on concentric rings with radii 2-mm; Pachy_Apex, corneal thickness in apex; Rv_B (mm), central radius in vertical direction (posterior); Rf_B (mm), flattest radius of posterior corneal curvature; Rf_F (mm), flattest radius of anterior corneal curvature; C.Vol_D_3mm, corneal volume at 3-mm; D8mm_Prog, pachymetric progression in an 8-mm diameter zone around the cornea's thinnest point; R_Per_F (mm), average anterior radius of curvature between 6- and 9-mm zone

Applying ANOVA to the refined set of 32 features allowed for the ranking of these features based on their variance scores (Fig. 2). This analysis highlighted the features that contribute most significantly to differentiating between the categories of NKC, SCKC, and KC corneas. As illustrated, the top five features/indices identified are: keratometry of the steepest point, anterior (Kmax (D)), index of surface variance (ISV), index of vertical asymmetry (IVA), index of height decentration (IHD), and index of height asymmetry (IHA). The details and significance of these features in classifying corneas will be described later in the Discussion section.

### Balancing class sampling

The raw dataset exhibits an imbalanced distribution across the different diagnostic classes. Specifically, there are significantly more samples in the NKC category compared to others, which is a common challenge in medical research. This imbalance can lead to biased classification outcomes, where ML models may perform well on the majority classes but poorly on the minority classes. Such biases can skew the model’s performance, potentially leading to less accurate and less reliable diagnostic predictions for underrepresented conditions.

**Fig. 3:**
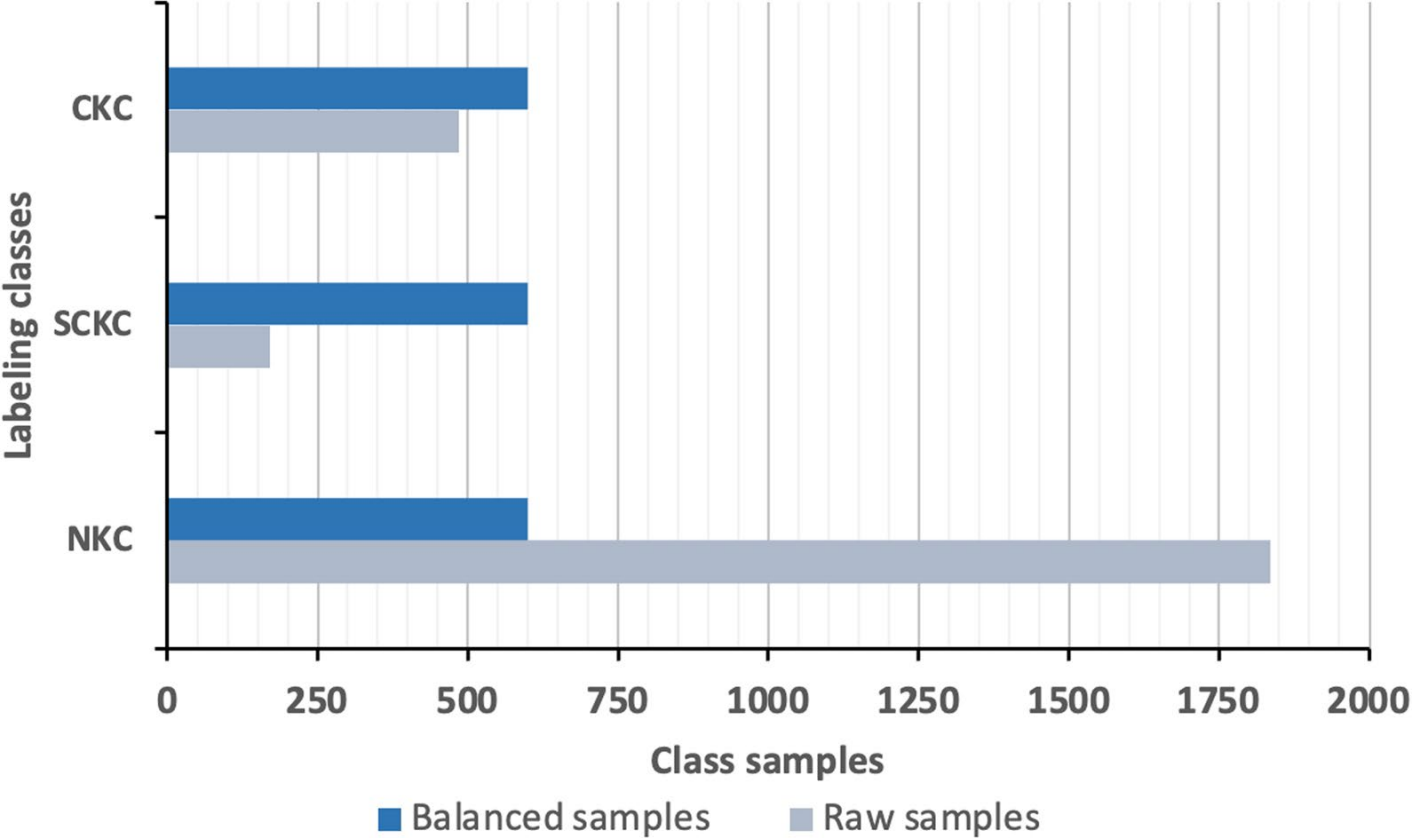
Comparison between the collected raw samples and balanced samples. CKC, clinical keratoconus; SCKC, subclinical keratoconus; NKC, non-keratoconus

To address the uneven class sampling distribution in the dataset, various methods can be employed, including oversampling the minority classes, under-sampling the majority classes, or implementing a combination of both approaches. These techniques aim to balance the dataset and enhance the performance of ML models. Given that the skewness ratio between the smallest and largest classes in the study dataset is relatively high (6.9:73.7), employing the latter approach is deemed the most appropriate option. As a compromise, the majority class of NKC corneas was down sampled from 1836 to 600 samples while augmenting the SKC and KC classes to match the new samples of majority class. The trimmed data samples from the NKC cornea class were carefully selected to ensure that valuable samples close to the decision boundaries were not lost. In contrast, each of the SCKC and CKC samples was augmented to 600 samples, using a synthetic minority oversampling technique (SMOTE). Figure 3 shows a comparison between the sample counts of the balanced dataset and the raw dataset. As illustrated, the balanced dataset now contained a total of 1800 samples, evenly distributed among the NKC, SCKC, and CKC classes.

SMOTE generated new data points along the line segments between a randomly selected data point and its nearest neighbors. By interpolating between existing instances of the minority class, SMOTE increased the representation of these classes in the dataset. This technique was employed in this study due to its effectiveness in addressing class imbalances, especially in small datasets [63–65]. These modifications were expected to improve the training and classification performance of the models while addressing the issues of small sample sizes.

### Machine learning modeling

Following the preparatory stages of pre-processing and feature selection, this section elaborates on the development of eight base models including RF, GB, DT, SVM, KNN, LDA, LoR, and naive bayes (NB), as well as several stacking ensemble combinations of these models. This process underwent unified training and hyperparameter tuning of these models. Since the development details of the base models were previously reported in [39], this section focuses on the proposed stacking ensemble models. However, the classification performance of both the base models as well as the stacking combinations of these models are evaluated and compared later in the Results section. The objective of these experiments was to identify the best performing stacking ensemble model for KC classification.

#### The stacking ensemble model

**Fig. 4:**
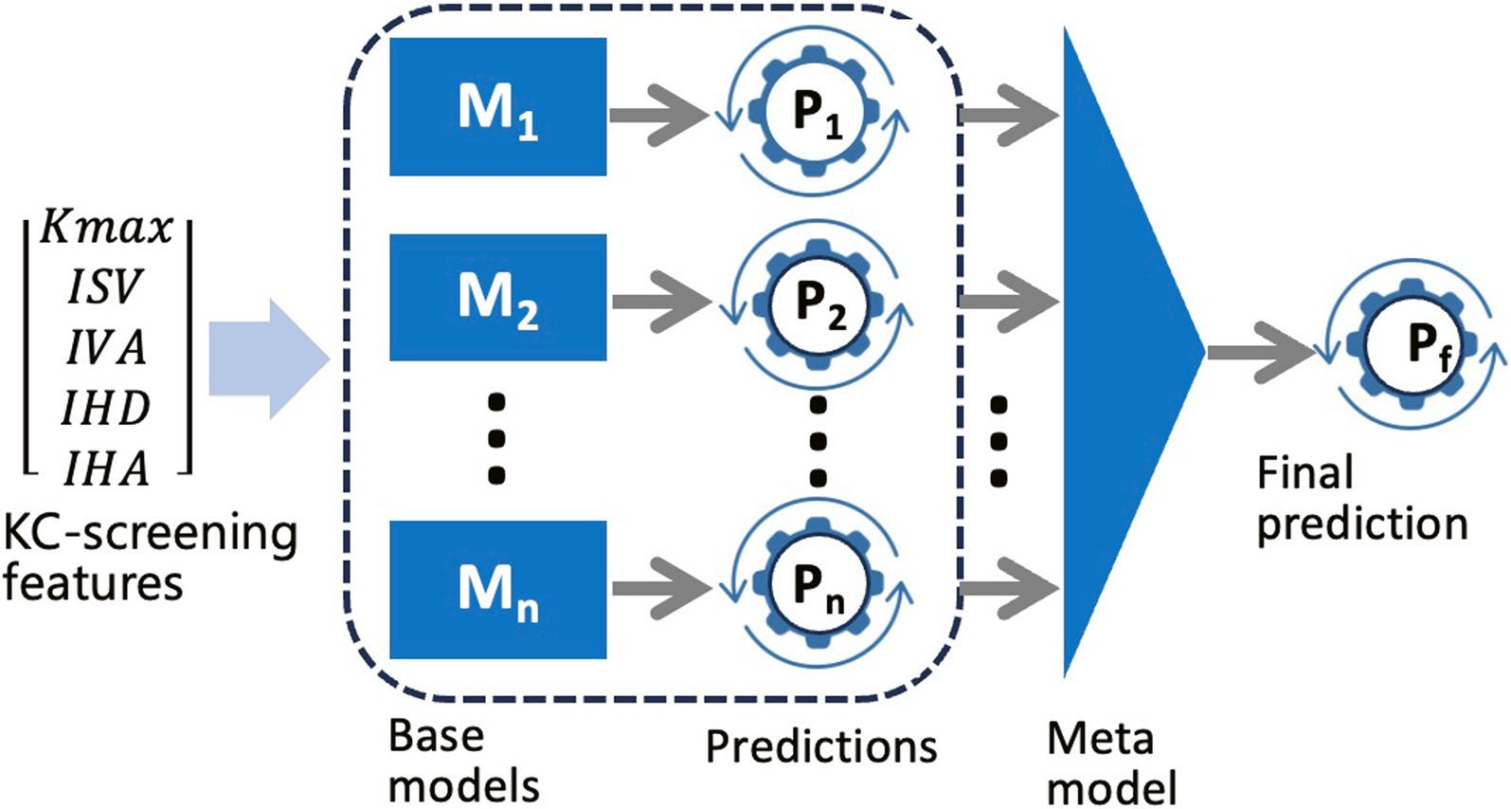
Framework of the proposed model learning approach

The proposed model in this study employed a stacking architecture consisting of two layers: Level 0 base models and a Level 1 meta-model. Figure 4 presents a simplified framework of this stacking ensemble learning approach. A series of stacking models were developed and evaluated to determine the best-performing configuration. These models were built using combinations of eight base learners: RF, GB, DT, SVM, KNN, LDA, LoR, and NB. The individual performance of each base learner within the stacking models had been previously assessed [39].

At Level 0 of the model, the base learners were trained using a cross-validation technique, which ensured that the entire training dataset was utilized effectively. Cross-validation splits the data into multiple subsets, known as 'folds,' with each base learner trained on different combinations of these subsets and validated on the remaining fold. This process reduced the risk of overfitting and ensured a more generalized model. The predictions generated by the base learners during cross-validation were then aggregated to form a new feature matrix, which served as input for the Level 1 meta-classifier.

In the Level 1 meta-model layer, a secondary model was trained to make the final predictions. This layer took the predictions from the base learners as input features and selected the most suitable classifier to combine and refine these predictions for the final output. The meta-classifier was designed to capture and exploit patterns in the relationships between the base learners’ predictions, further improving overall predictive performance by addressing any weaknesses or biases in the individual models. This layered approach enhanced the model’s ability to generalize and deliver more accurate final predictions.

Unlike voting, where the final prediction is made by either selecting the most frequent class among the classifiers or weighting their predictions, the proposed model combined these baseline predictions through a meta-classifier or blender. The key idea behind the Level 1 meta learner was to capitalize on the strengths of the base models while addressing their individual limitations, thereby generating more accurate and robust predictions by appropriately weighing their outputs. The predictions from the base models were then combined to make predictions on the validation set.

#### K-folding and training

**Fig. 5:**
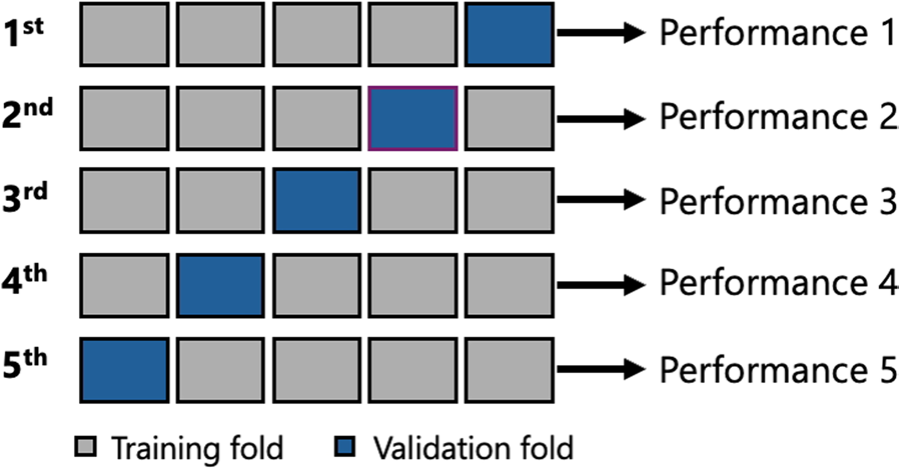
Schematic of the five-fold cross validation process

Each of the developed models was trained using K-fold cross-validation, where the dataset was split into K equal folds. In each iteration, one of the K-folds was set aside as the validation set, while the remaining K-folds were utilized for training. This process allowed for a comprehensive evaluation of the model's performance across different subsets of the data. In this study, five-fold cross-validation was implemented, with four folds (80%) allocated for training and the remaining fold (20%) used for validation, as illustrated in Fig. 5. The model's performance was evaluated by averaging the metrics—such as accuracy, precision, recall, and others—across all iterations. This approach provided a robust and more accurate estimate of the model's overall performance compared to a single train-test split. The average performance is calculated using Eq. (1), as follows:

1 $$Performance (ave) =\frac{1}{5} \sum_{i=1}^{5}Performance (i)$$

#### Hyperparameter tuning

Choosing optimal hyperparameters is a key challenge in ML development as they define model settings and greatly influence the learning process. Identifying the best settings typically requires iterating through various options to assess their impact on accuracy. In this study, two Scikit-learn methods—GridSearchCV (GSCV) and RandomSearchCV (RSCV)—were used to optimize the hyperparameters of all models examined. GSCV conducted an exhaustive search over a predefined set of hyperparameter values within a specified range, systematically evaluating every possible combination to identify the best-performing model [66, 67]. In contrast, RSCV assigned values to each hyperparameter based on a probability distribution, randomly selecting combinations to test. This randomized approach significantly reduced computational time, making RSCV faster and more efficient than GSCV, especially when dealing with large hyperparameter spaces [68]. Nevertheless, results from numerous experiments indicated that the hyperparameters tuned using GSCV outperformed those obtained by RSCV, leading to the adoption of GSCV in this study despite its slower performance. The hyperparameters of the top-performing models are presented in the Appendix, along with the meta-model, used to build the proposed stacking ensemble.

### Clinical classification

The classification of corneas into NKC, SCKC, and CKC was based on a comprehensive ophthalmic evaluation conducted by experienced ophthalmology specialists. This evaluation followed a stepwise approach, beginning with an optometric assessment to determine refractive error. Subjective refraction was performed to gauge the individual's perception of optimal visual correction, while objective refraction was assessed using retinoscopy. The best-corrected visual acuity (BCVA) was measured to evaluate the maximum visual clarity achievable with corrective lenses. Following these assessments, the cornea was closely examined using a slit lamp and interpretation of Pentacam corneal tomography images, which provided detailed insights into the corneal structure and shape.

For corneas to be classified as NKC, several criteria needed to be met. Firstly, the Pentacam corneal tomography had to show no abnormalities, particularly no signs of irregular corneal surfaces or suspicious patterns in the curvature, elevation, or thickness maps. The BCVA in these corneas was required to be 20/20, indicating perfect or near-perfect visual acuity. Additionally, the slit-lamp examination had to show no clinical signs of KC, such as corneal thinning, distortion, or scarring. Furthermore, both subjective and objective refraction should show no evidence of irregular astigmatism, a key indicator of abnormal corneal shape.

Corneas classified as having SCKC demonstrated normal or near-normal clinical findings but exhibited subtle abnormalities on corneal tomography. In these cases, the BCVA remained 20/20, and the slit-lamp examination was largely unremarkable, potentially revealing only minimal signs that might suggest early KC. Despite this, advanced corneal imaging with the Pentacam detected early-stage irregularities, including increased posterior corneal elevation, localized thinning in the corneal pachymetry map, or steepening of the anterior corneal surface. These findings were frequently associated with the asymmetry between the anterior and posterior corneal surfaces. Key Pentacam indices such as the IVA, ISV, and IHD were typically elevated, indicating subtle but measurable distortions in corneal shape that are not detectable through standard clinical exams alone.

In contrast, corneas classified as having CKC exhibited clear and pronounced abnormalities both on clinical examination and corneal tomography. Tomographic maps showed marked distortions, with characteristic findings on the curvature, elevation, and pachymetry maps, such as significant steepening of the anterior corneal surface, increased posterior elevation, and focal thinning of the cornea. These corneas often had reduced BCVA due to the irregular shape of their cornea, which could no longer be fully corrected with standard lenses. Irregular astigmatism, a hallmark of KC, was commonly observed in both subjective and objective refraction assessments, alongside a scissoring reflex on retinoscopy. Slit-lamp examination often revealed additional clinical indicators of KC, such as corneal striae or scarring, particularly in more advanced cases. These findings reinforced the diagnosis of clinical KC, distinguishing these corneas from those with SCKC or NKC presentations.

This classification process enabled a clear and accurate differentiation between NKC corneas and those exhibiting varying stages of KC, ensuring precise diagnosis and tailored management for each group. By integrating detailed clinical assessments with advanced corneal imaging, this approach allowed for early detection of subtle abnormalities in subclinical cases and identification of more pronounced changes in CKC, optimizing patient care and treatment outcomes.

## Results

The experiment started with a raw clinical dataset of 2491 subjects. After data cleaning, the initial 79 feature columns were reduced to 58. A rigorous feature selection process followed, incorporating feature dependency analysis, expert input, and analysis of feature-target relationships. This process systematically reduced the feature set to a final subset of five, which was used to train and validate both the base and stacking models in this study. The impact of data cleaning and feature selection was crucial in improving model performance.

### Dataset balancing

**Fig. 6:**
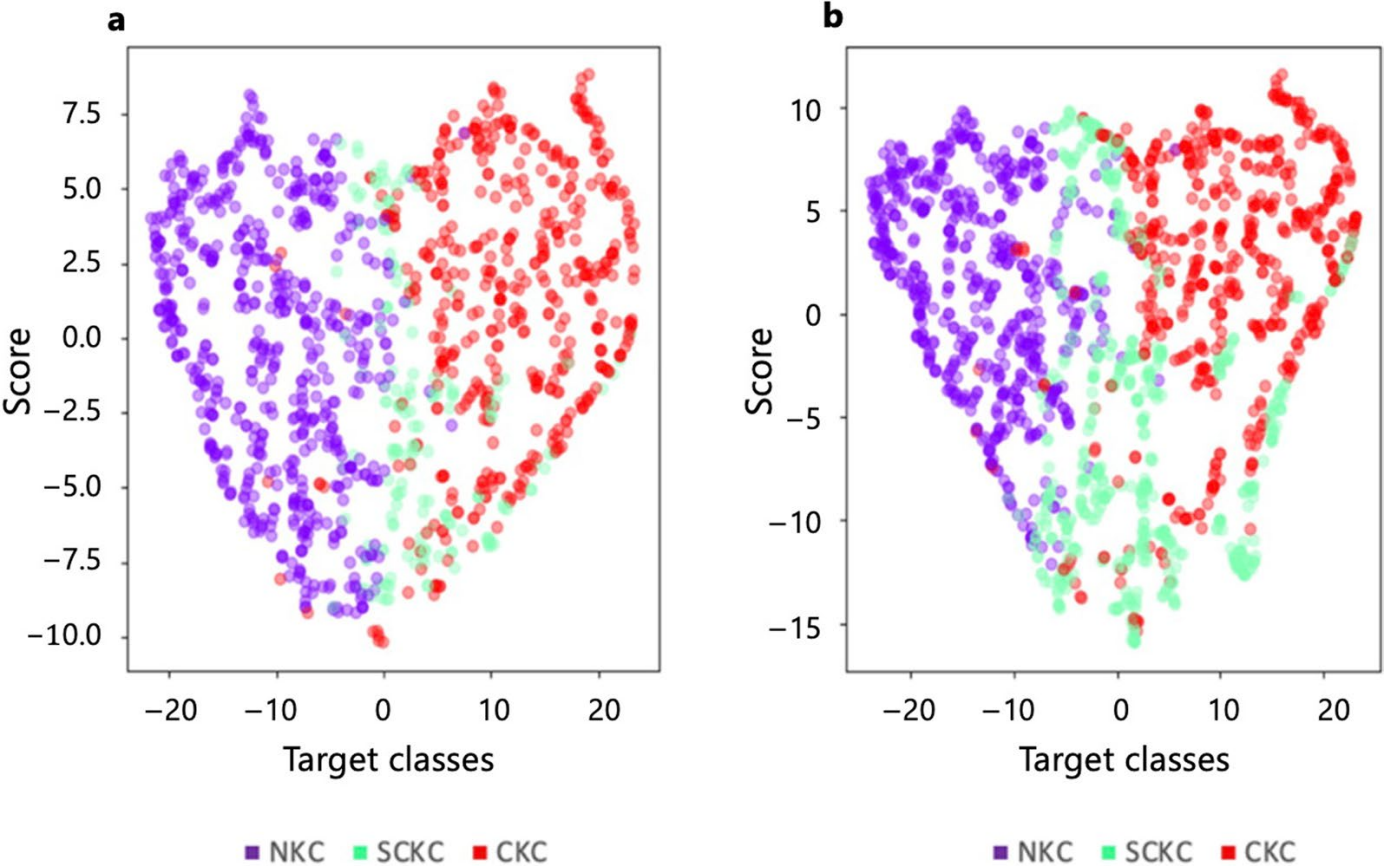
Class samples comparison before and after oversampling of the minority classes. **a** Before oversampling, ratio=13.6:47.8; **b** After oversampling, ratio=33.3:33.3. CKC, clinical keratoconus; SCKC, subclinical keratoconus; NKC, non-keratoconus

After trimming the majority class sampling of the NKC corneas to 600 samples, the samples for the SCKC and CKC corneas were augmented by synthesizing additional samples to match the number of samples in the majority class (NKC). This process ensured a more balanced representation of each class within the dataset. Figure 6 illustrates the class distribution before and after applying the oversampling technique. As depicted, the minority-to-majority class ratio improved significantly from 13.6:47.8 to an equitable 33.33:33.33. This adjustment not only reduced bias in the dataset but also led to enhanced model performance and improved generalization across all classes, allowing the models to better recognize and classify each category without being skewed toward the majority class.

### Feature selection

Feature selection is a vital step in the ML pipeline, playing a crucial role in creating models that are not only more efficient and accurate but also easier to interpret. In this study, the feature selection process involved a comprehensive analysis of feature-target relationships, feature dependencies, and variance. This approach integrated statistical methods with the expertise of ophthalmologists to ensure clinical relevance of the selected features. As a result of these techniques, the original dataset was systematically reduced from 79 features to a refined subset of just five, representing only 6.33% of the total features. Reducing the input features of a model without compromising performance is crucial for improving efficiency and reliability. This approach helps prevent overfitting, ensuring that the model captures only essential patterns rather than noise from excessive data. Additionally, using fewer parameters reduces computational costs, leading to faster training and inference times, which is particularly advantageous for large datasets and real-time applications.

The impact of feature selection on classification performance and training time was assessed experimentally by comparing the full 51-feature set with a refined 5-feature set. The results showed that the stacking model achieved a classification accuracy of 99.17% with all 51 features, compared to a higher 99.72% accuracy with the refined 5-feature set. Moreover, the training time for the full 51-feature set was 12.68 seconds, while it was reduced to 1.79 seconds with the 5-feature set (i.e., a computing efficiency gain of over 85%). Since training times vary by computing device, these experiments were conducted on a MacBook Air with an Apple M2 processor, 16 GB RAM, and macOS Sonoma.

### Hyperparameter tuning

**Fig. 7:**
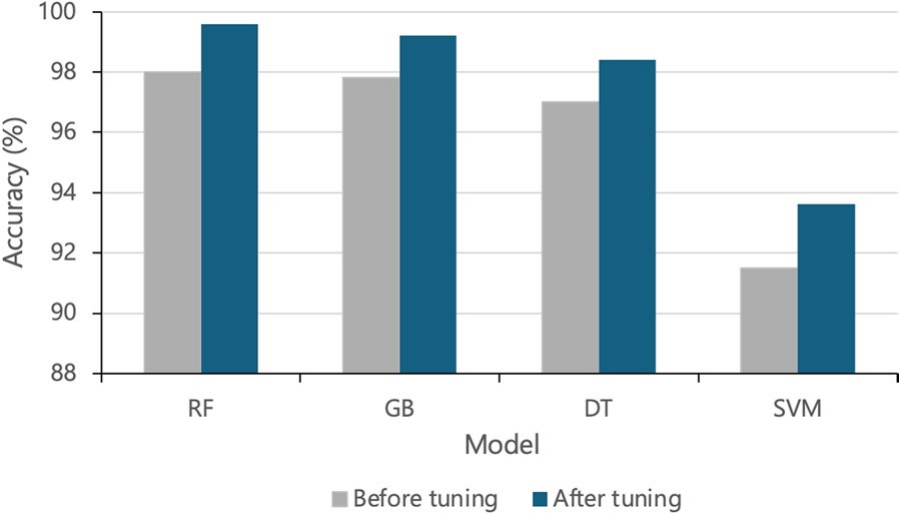
Performance comparison before and after hyperparameter tuning. RF, random forest; GB, gradient boosting; DT, decision tree; SVM, support vector machine

In this study, the hyperparameter tuning was conducted separately for each base model (RF, GB, DT, and SVM) before integrating them into the proposed stacking model. This optimization process improved classification accuracy, with fine-tuned models consistently outperforming those using default parameter settings. Figure 7 illustrates these performance gains across all models, highlighting the impact of hyperparameter optimization in maximizing the overall model effectiveness.

### Performance evaluation

A confusion matrix, a commonly used tool for evaluating classification model performance, was employed to assess the effectiveness of various stacking model combinations. This matrix visually compares predicted and actual class labels, with the ground truth (actual target classes) plotted along the x-axis and predicted classes displayed on the y-axis. True positives (TPs) occur when the model correctly predicts a positive class (both actual and predicted values are 1), while true negatives (TNs) indicate both predicted and actual values are 0. False negatives (FNs) occur when the model predicts 0 for an actual class value of 1, while false positives (FPs) arise when the model predicts 1 for an actual class value of 0. After conducting extensive experiments with the base models, the performance of each model was thoroughly evaluated using their corresponding confusion matrices. From these matrices, several standard performance metrics were derived, including accuracy, precision, sensitivity (recall), F1-score, and F2-score. These metrics were calculated using Eqs. (2, 3, 4, 5 and 6) [3], as follows:

2 $$Accuracy=\frac{TP+TN}{TP+TN+FP+FN}$$

3 $$Precision=\frac{TP}{TP+FP}$$

4 $$Sensitivity \left(or Recall\right)=\frac{TP}{TP+FN}$$

5 $${F1 {\text{-}} score} =2\times \left(\frac{Precision\times Sensitivity}{Precision+Sensitivity} \right)$$

6 $${F2 {\text{-}} score} = 5\times \left(\frac{Precision\times Sensitivity}{\left(4\times Precision\right)+Sensitivity}\right)$$

**Fig. 8:**
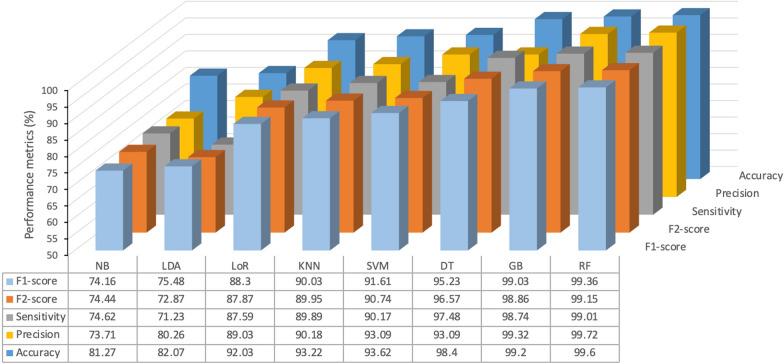
Performance comparison of the examined base models. NB, naive Bayes; LDA, linear discriminant analysis; LoR, logistic regression; KNN, K-nearest neighbor; SVM, support vector machine; DT, decision tree; GB, gradient boosting; RF, random forest

Unlike the F1-score, which gives equal weight to precision and sensitivity, the F2-score reduces the importance of precision while placing greater emphasis on sensitivity. This makes it more focused on minimizing FN rather than FP. Figure 8 provides a performance comparison of the evaluated base models. The results clearly demonstrate that the tree-based models—RF, GB, and DT—consistently outperformed other models, followed closely by SVM model.

**Fig. 9:**
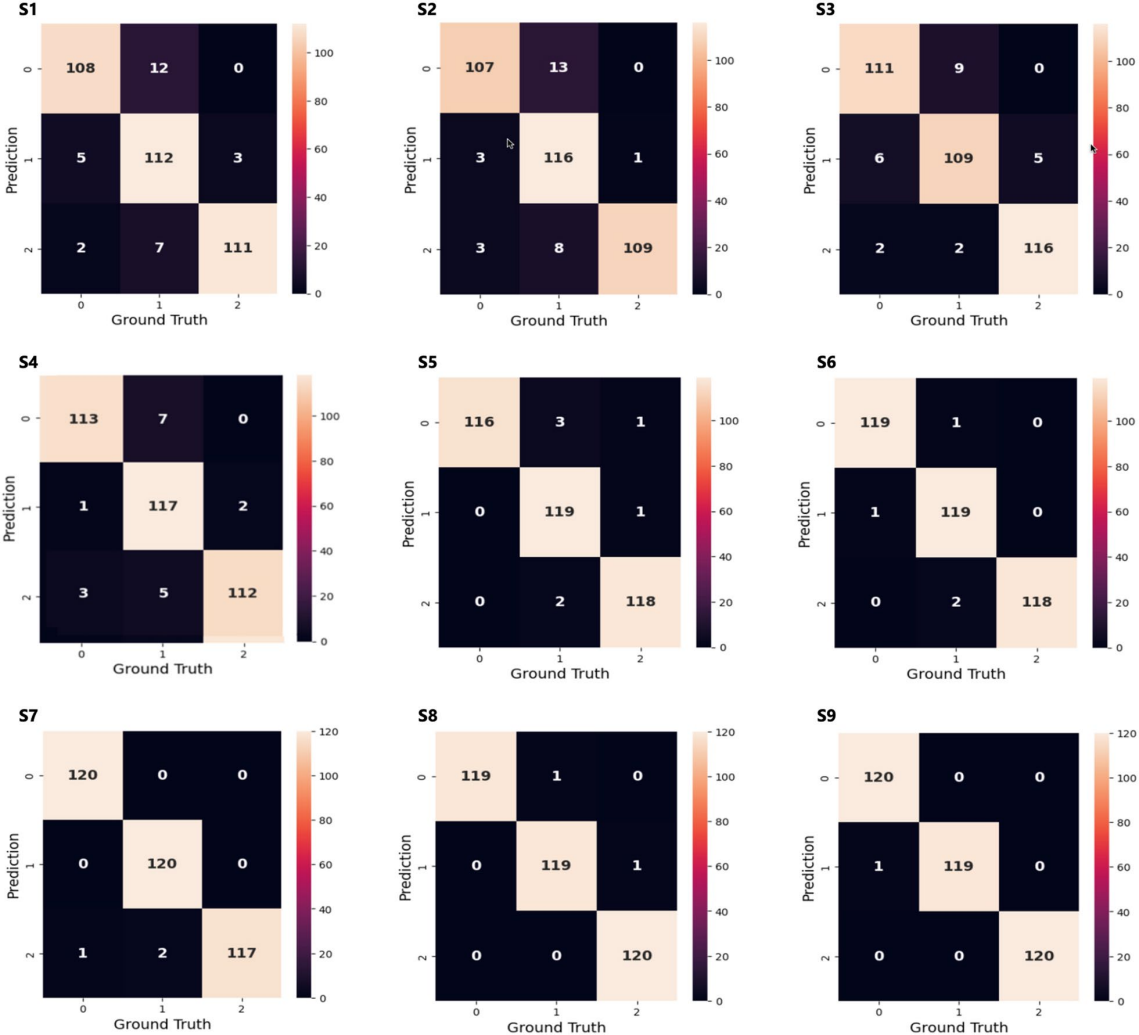
Confusion matrices for representative examples of the examined stacking ensemble learning models. **S1** (naive bayes, K-nearest neighbors, linear discriminant analysis); **S2** (logistic regression, linear discriminant analysis, naive bayes); **S3** (linear discriminant analysis, K-nearest neighbors, logistic regression); **S4** (support vector machines, naive bayes, logistic regression); **S5** (gradient boosting, support vector machines, K-nearest neighbors); **S6** (naive bayes, logistic regression, gradient boosting); **S7** (random forest, decision trees, K-nearest neighbors); **S8** (random forest, support vector machines, decision trees); **S9** (random forest, gradient boosting, decision trees)

A similar performance evaluation process was applied to the various stacking model combinations to identify the best-performing ensemble. Figure 9 presents representative examples of confusion matrices from these stacking experiments. The numerical values (0, 1, and 2) shown on the bottom and left sides of each matrix correspond to the class labels for NKC, SCKC, and CKC, respectively. In all the stacking configurations, The SVM model was selected as the meta-model responsible for generating the final classification results. This decision stemmed from extensive experimentation with various base model combinations, where SVM consistently showed superior performance in refining and integrating predictions. Using SVM as the meta-classifier improved classification accuracy across the three diagnostic classes.

This multi-metric evaluation approach facilitated a deeper understanding of the models' strengths and weaknesses, providing a comprehensive assessment of their performance. By employing a range of metrics, we were able to capture various aspects of classification performance, including accuracy, precision, sensitivity (or recall), and F1-score. This balanced comparison highlights not only the overall effectiveness of each model but also specific areas for improvement, allowing for a more informed selection of the best-performing models. Ultimately, this thorough evaluation ensured that the chosen model was robust and well-suited for the task at hand.

**Fig. 10:**
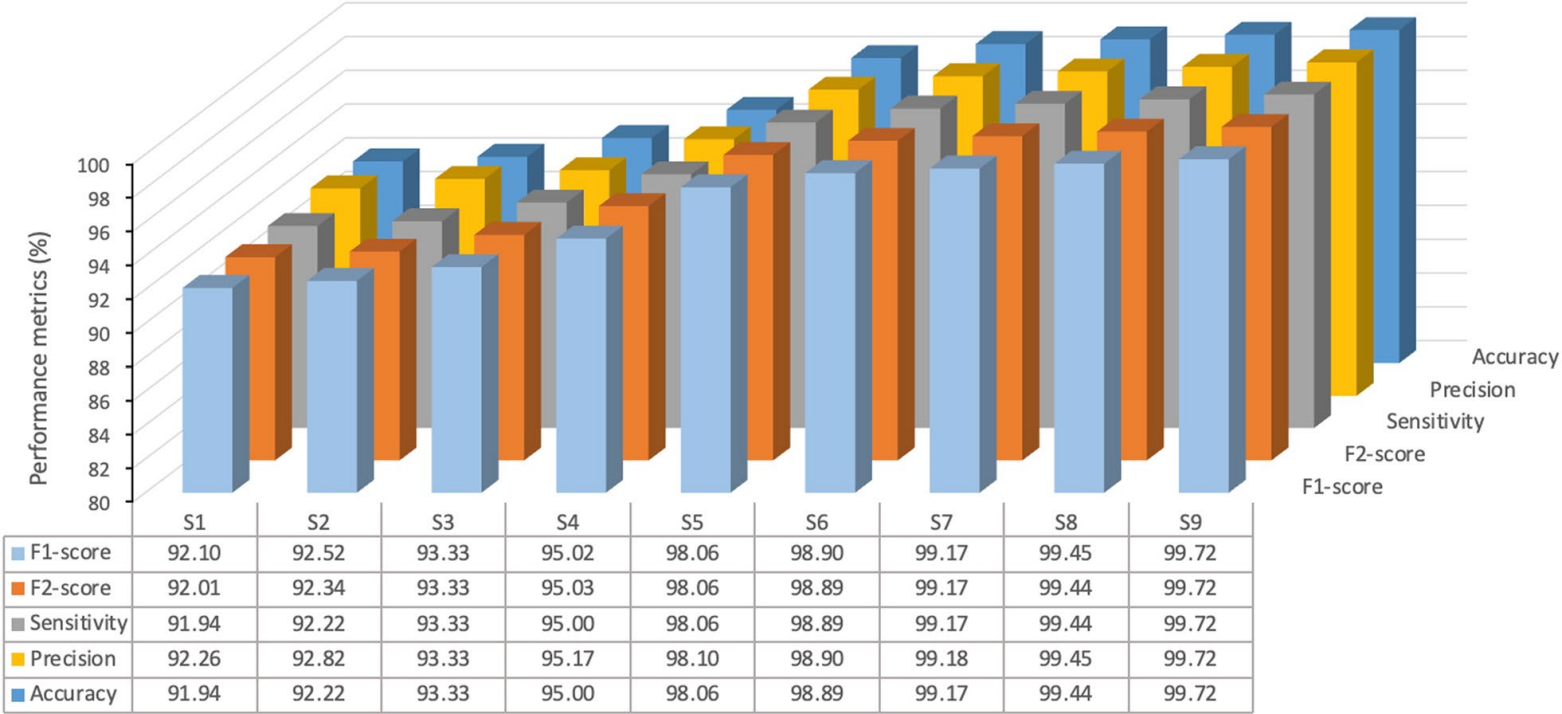
Performance comparison for examples of stacking ensemble learning models. S1 (naive bayes, K-nearest neighbors, linear discriminant analysis); S2 (logistic regression, linear discriminant analysis, naive bayes); S3 (linear discriminant analysis, K-nearest neighbors, logistic regression); S4 (support vector machines, naive bayes, logistic regression); S5 (gradient boosting, support vector machines, K-nearest neighbors); S6 (naive bayes, logistic regression, gradient boosting); S7 (random forest, decision trees, K-nearest neighbors); S8 (random forest, support vector machines, decision trees); S9 (random forest, gradient boosting, decision trees)

Figure 10 provides a detailed performance comparison of the stacking models corresponding to the confusion matrices presented in Fig. 9. As shown, the performance varied based on the specific combinations of base models employed. Notably, stacking models that incorporated tree-based algorithms—specifically RF, GB, and DT— consistently outperformed other model stacking configurations. This enhanced performance highlights the ability of tree-based models to capture intricate patterns and interactions within the corneal data, making them particularly well-suited for the task. The results suggest that such algorithms have strong potential for real-world clinical applications in KC diagnosis where reliable and accurate detection is critical. This improved performance underscores the ability of tree-based models to capture complex patterns and interactions within the corneal data, making them particularly well-suited for the task of KC detection. The results suggest that these algorithms hold significant potential for real-world clinical applications where reliable and accurate diagnosis is paramount.

To further validate the effectiveness of the models, an additional performance metric—Matthews correlation coefficient (MCC)—was employed. The MCC is particularly valuable for evaluating the quality of classification models because it considers all elements of the confusion matrix: TP, TN, FP, and FN. This makes MCC a robust and balanced metric, especially in cases of imbalanced datasets its value ranges from −1 (indicating total disagreement between predicted and actual labels) to +1 (indicating perfect classification), with 0 representing a random or no better-than-chance classifier [69]. The MCC was calculated using Eq. (7), as follows:

7 $$MCC = \frac{\left(TP\times TN\right)-(FP\times FN )}{\sqrt{TP+FP) \times \left(TP+FN\right) \times (TN+FP) \times \left(TN+FN\right)}}$$

**Fig. 11:**
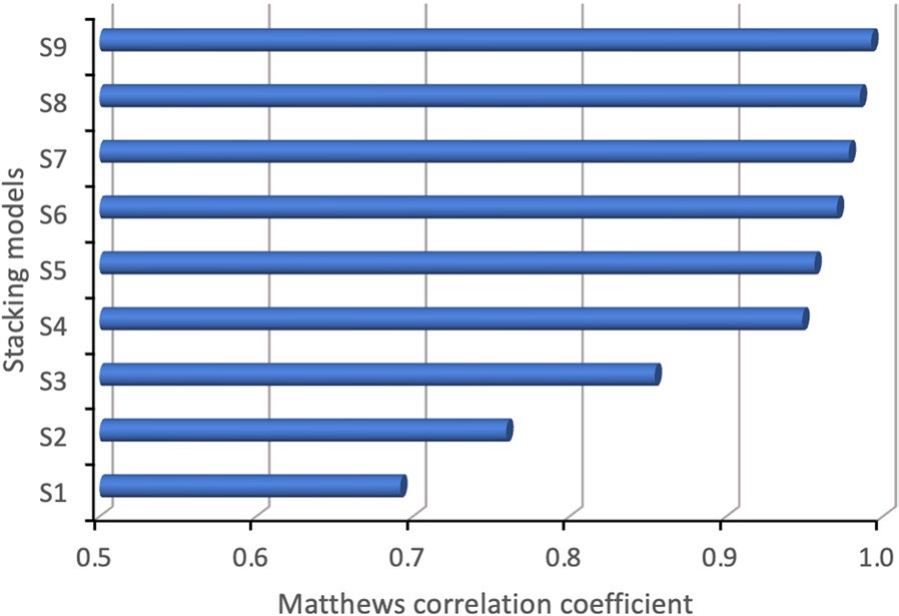
Matthews correlation coefficient of the stacking ensemble learning models

Figure 11 displays the MCC values for the evaluated stacking models. As shown, the stacking model S9—comprising RF, GB, and DT as base classifiers, with SVM as the meta-classifier—outperforms all other models, achieving an outstanding MCC score of 0.995. These results highlight the superior classification performance of tree-based algorithms, especially when used in ensemble configurations, further solidifying their effectiveness in handling the complexities associated with KC detection.

## Discussion

This section discusses the selected feature subset, the classification performance of the developed stacking model, and a comparison of its performance with existing methods.

### The selected feature subset

The key feature subset used to distinguish between NKC, SCKC and CKC corneas is outlined briefly as follows [70–73]:

*Kmax (D):* The refractive power of the steepest meridian of the cornea (i.e., the highest axis of corneal power). This feature measures the maximum curvature at the anterior corneal surface, which is crucial in classifying clinical KC corneas into mild, moderate and severe [74].

*ISV:* The standard deviation of individual corneal sagittal radii from the mean sagittal curvature. Values greater than 37 are suspicious for SCKC, this index assesses the variability in corneal surface curvature, which is sensitive to monitor SCKC and CKC progression.

*IVA:* This index quantifies the average disparity between superior and inferior corneal curvature (expressed in mm) i.e., the asymmetry of the corneal curvature in respect to the horizontal meridian. Values greater than 0.28 mm are suspicious. This index helps to detect irregular patterns associated with SCKC.

*IHD:* The measurement of elevation data offset in the vertical direction. It is a measure of the degree of decentration of the corneal elevation map. This index is relevant for detecting KC, values greater than 0.014 μm should raise the suspicion of SCKC.

*IHA:* The average deviation of corneal height measurements on tomography elevation maps from the horizontal meridian—specifically, the variation between the superior and inferior corneal elevations—is a highly sensitive parameter for detecting SCKC. Values greater than 19 μm are considered abnormal, indicating potential early-stage corneal irregularities [75].

These corneal characteristics, along with a few others, have been widely recognized as reliable parameters for detecting KC, and SCKC specifically [76, 77]. These technical findings also align well with the diagnostic criteria employed by ophthalmologists at the Jordanian eye-care facilities. Other indices, such as the KC index (KI) and central KC index (CKI), though mentioned in these studies, showed lower variance scores and were thus excluded from the final selection. From a technical standpoint, the reduction in the feature subset not only streamlined the model but also brought several key advantages. By decreasing the number of input features, the overall complexity of the model was significantly reduced, making it more computationally efficient. This led to faster training times, which are especially beneficial when working with large datasets or in situations where rapid model iterations are necessary.

### Classification performance

Despite utilizing a relatively small input feature set for classifying NKC, SCKC, and CKC corneas, the proposed stacking model—combining the strengths of top-performing base classifiers (RF, GB, and DT) with SVM as the meta-classifier—outperformed both its individual components and all other stacking combinations explored in this study across all evaluation metrics. It achieved accuracy, precision, sensitivity, F1 and F2 scores of 99.72% and MCC score of 0.995, indicating nearly perfect performance. As shown in the confusion matrix in Fig. 9 (S9), the model correctly classified all NKC and CKC test cases, with only a single misclassification in an SCKC test case. Error analysis revealed that this misclassified case had borderline parameters, emphasizing the need for additional training data around the transition boundaries between different conditions. The individual base models that make up the stacking model also misclassified this case, along with additional SCKC and NKC cases. Notably, all models perfectly classified all CKC cases. More importantly, unlike the individual models, the stacking model exhibited greater robustness and performance consistency when tested on an additional dataset of 100 unseen cases.

In contrast, the stacking combination of NB, KNN, and LDA (referred to as S1) resulted in the lowest performance, primarily due to the exclusion of any top-performing algorithms. This combination achieved accuracy and sensitivity of 91.94%, indicating that while it could correctly predict a substantial portion of the instances, it fell short of the benchmarks set by other models. Furthermore, S1 recorded the lowest classification quality score of just 0.88, underscoring its overall lack of robustness compared to other combinations. Other stacking models exhibited varying levels of performance based on the specific base models used, highlighting the significant impact of model selection on classification outcomes. This performance variability highlights the importance of selecting high-performing base models to improve the overall effectiveness of the stacking approach.

### Performance comparison

A performance comparison between the proposed model and existing methods in the literature that rely exclusively on base models is provided in Table 2. This comparison considers common performance metrics as well as other relevant factors, such as dataset availability (local or public), corneal imaging devices used, and sizes of the input feature set. The most recent and closely comparable performance to the proposed stacking model was previously reported [39]. They used an RF classifier with a five-feature input set, achieving 99.6% accuracy, 99.01% sensitivity, 99.72% precision, and F1 and F2 scores of 99.63 and 99.15, respectively. The second-best results were reported in [35, 38], both using RF classifiers with input sets of 10 and 15 features, respectively. The first study achieved 98% accuracy, sensitivity, and precision, while the second study achieved the same accuracy and precision but with a slightly lower sensitivity of 96%.

**Table 2:** As of 2019, performance comparison of the proposed model with the existing state-of-the-art methods

Authors	Model used	Imaging device used	Dataset used			Performance metrics (%)		Input feature set
			Availability	Feature set	Subjects used	Accuracy	Other metrics	
Issarti et al. [28]	FNN	Pentacam	Local	5	851	96.56	Sen: 97.78; Spe: 95.56	5
Shi et al. [29]	ANN + others	Pentacam + UHR-OCT	Local	49	121	n/a	Sen: 98.5; Spe: 94.7; AUC: 93	49
Lavric et al. [30]	SVM	CASIA SS-1000 (OCT)	Public	8	3151	94	Sen: 87; Spe: 98	8
Cao et al. [34]	RF	CASIA SS-1000 (OCT)	Local	11	88	97	Sen: 94; Spe: 90	5
Aatila et al. [35]	RF	CASIA SS-1000 (OCT)	Public	446	3162	98	Sen: 98; Pre: 98	10
Shanthi et al. [31]	SVM	Pentacam	Local	31	205	91.8	Sen: 94.2; Spe: 97.5	5
Malyugin et al. [32]	QDA	Pentacam	Local	490	47,419	n/a	AUC: 95	7
Herber et al. [36]	RF	Pentacam	Local	23	434	78	Sen: 80; Spe: 90	10
Castro-Luna et al. [37]	RF	Pentacam	Local	81	81	89	Sen: 86; Spe: 93	n/a
Cao et al. [38]	RF	Pentacam	Local	267	1692	98	Sen:96; Spe: 98	15
Priya et al. [78]	SVM lePara>	CASIA SS-1000 (OCT)	Public	447	3164	93.3	Pre: 94.1; Spe: 97.7	2
Song et al. [33]	DT	BCT scan	Local	194	194	92.4	Sen: 90.3; Spe: 94.3	20
Muhsin et al. [39]	RF + others	Pentacam	Local	79	2491	99.6	Sen: 99.01; Pre: 99.72; F1: 99.63; F2: 99.15	5
Proposed stacking model	Stacking (RF, GB, DT + SVM meta classifier)	Pentacam	Local	79	2491	99.72	Sen: 99.72; Pre: 99.72; F1: 99.72; F2: 96.88; MCC: 99.6	5

*ANN* = artificial neural network; *BCT= * biomechanical computed tomography; *DT* = decision tree; *FNN* = feedforward neural network; *GB* = gradient boosting; *QDA* = quadratic discriminant analysis; *RF* = random forest; *SVM* = support vector machine; *AUC* = area under the curve; *F1* = F1-score; *F2* = F2-score; *Pre* = precision; *Sen* = sensitivity; *Spe* = specificity; *MCC* =Matthew’s correlation coefficient; *n/a* = not applicable

For this comparison, we focused on studies that addressed similar KC classification conditions (NKC, SCKC, and CKC). We excluded studies that dealt with binary classifications (e.g., NKC vs. CKC, NKC vs. SCKC, or SCKC vs. CKC), CKC severity staging, or those that used models with image inputs. In studies evaluating multiple base models, only the best-performing model is given in Table 2. It is also important to acknowledge the inherent challenge of making direct comparisons between studies due to the absence of a standardized grading system for KC detection. Each study may use different diagnostic criteria, tools, and thresholds, resulting in variations in how the disease is identified and graded. Additionally, datasets can vary in terms of sample size, demographics, and the conditions of KC being evaluated. These inconsistencies complicate the comparison of findings across studies and limit the ability to draw universal conclusions about the efficacy of diagnostic methods or treatment outcomes for KC detection. Establishing a uniform grading system and standardized datasets would greatly enhance the comparability and reliability of future research in this field.

Given its superior and robust performance, the developed stacking model holds significant promise for enhancing the clinical practice of KC screening by:

- Promoting the use of a standardized, objective diagnostic method for KC detection among eye-care professionals. This will minimize variability in diagnoses and ensure consistent patient management across various clinical settings, leading to more reliable outcomes and improved continuity of care.
- Expanding accessibility to KC diagnosis across a wide range of eye-care facilities by deploying the developed stacking model in a web-based application. This will enable practitioners to access the diagnostic tool from any location at any time, enhancing flexibility in patient care.
- Utilizing measurements from a single corneal imaging device, the model ensures compatibility and ease of use, streamlining the diagnostic process, reducing cost, and making KC detection more widely available, even in remote or resource-limited settings.
- Providing automated analysis, which is particularly crucial in areas with limited access to expert ophthalmologists. This would also help bridge the gap in areas where specialized expertise is scarce, enabling more accurate and timely KC screening.
- Assisting ophthalmologists by providing reliable, data-driven insights, enhancing decision-making in settings where interpreting advanced diagnostic imaging may be challenging. By reducing reliance on subjective assessments, a KC screening tool based on the developed model promotes consistency and improves diagnostic confidence, even in under-resourced healthcare environments.

Since KC is a relatively rare disease, collecting a large dataset was challenging, particularly for SCKC and CKC cases. To partially address this, synthetic data were generated for these underrepresented classes. However, despite these efforts, there remains a critical need for continued data collection to enhance the model’s robustness. Additionally, testing with unseen data was limited to 100 cases, restricting a comprehensive evaluation of the model’s generalizability. To mitigate this limitation, future iterations of the model will prioritize acquiring larger and more diverse datasets to improve its adaptability for real-world clinical use.

## Conclusion

The ongoing evolution of corneal imaging modalities and ML diagnostic methodologies holds the potential to significantly enhance our understanding and management of KC, leading to more comprehensive diagnostic approaches and improved patient care. As ML techniques continue to progress, they offer promising avenues for refining diagnostic precision, identifying subtle KC patterns, and facilitating personalized treatment strategies tailored to individual patient needs. These advancements are pivotal in advancing the field of ophthalmology, aiming to optimize early detection, intervention, and long-term KC management for better clinical outcomes. Collaboration between ML experts and ophthalmologists is crucial for advancing clinical practice and enhancing diagnostic capabilities.

To improve the KC diagnostic process, we have developed a highly efficient decision support model designed specifically for KC screening. This collaborative approach not only refines the diagnostic process but also helps bridge the gap between advanced ML methods and practical, real-world applications in ophthalmology. A reliable subset of corneal indices was identified using various statistical and visual techniques and validated by a team of expert ophthalmologists. Despite its compact size, this highly effective set of indices was used to train and validate an advanced stacking ensemble learning model. The selected input feature set simplified the model's structure and significantly reduced training time, all while maintaining near-perfect predictive performance. The findings reported in this study indicate that ML holds significant promise for enhancing KC screening and improving patient care in routine ophthalmologic practice.

Future improvements of the developed model will focus on the following key areas:

- Integrating the model into a web application, providing ophthalmologists with seamless access across diverse eye-care settings. This integration will facilitate additional data collection and enable further functional, acceptability, and usability testing through a pilot study with additional unseen data.
- Automating the transfer of corneal measurements from Pentacam to minimize the potential human error and ensure data integrity.
- Expanding functionality to allow the web application to provide treatment recommendations and referral guidelines based on diagnostic outcomes.

These developments, among others, represent ongoing research efforts by the authors.

## Supplementary Information

Additional file 1

## Data Availability

The dataset analyzed in this study is not publicly accessible due to privacy regulations set by the collaborating institutions. However, it can be requested from the corresponding author with valid reasons.

## Abbreviations

ANN: Artificial neural network
AUC: Area under the receiver operating characteristic curve
BCVA: Best-corrected visual acuity
CKC: Clinical keratoconus
CNN: Convolutional neural networks
DT: Decision tree
FNN: Feedforward neural network
FN: False negative
FP: False positive
IHA: Index of height asymmetry
IHD: Index of height decentration
ISV: Index of surface variance
IVA: Index of vertical asymmetry
KC: Keratoconus
Kmax: Keratometry of the steepest point (anterior)
KNN: K-nearest neighbor
LoR: Logistic regression
MCC: Matthews correlation coefficient
ML: Machine learning
NB: Naive Bayes
NKC: Non-keratoconus
QDA: Quadratic discriminant analysis
RF: Random forest
SCKC: Subclinical keratoconus
SVM: Support vector machine
TN: True negative
TP: True positive
