# Supplementary material for: Highly efficient stacking ensemble learning model for automated keratoconus screening
Source: Eye Vis (Lond). 2025 Jun 24;12:25. doi: 10.1186/s40662-025-00440-6 (PMC12186405; doi:10.1186/s40662-025-00440-6)
Supplement: Supplementary file 1 — Additional file 1 [file 40662_2025_440_MOESM1_ESM.docx]

**Supplementary Table S1**. Hyperparameters of the proposed stacking ensemble model

| Model | Hyperparameter values/description |
| --- | --- |
| Random Forest (RF) | n_estimators = 150 criterion = squared_error max_depth = 8 min_samples_split = 2 min_samples_leaf = 2 |
| Gradient Boosting (GB) | n_estimators = 150 max_depth = 5 min_samples_split = 2 min_samples_leaf = 2 |
| Decision Tree (DT) | max_depth = 5 min_samples_leaf = 2 min_samples_split=5 colsample_bytree = 0.96 |
| Support Vector Machine (SVM) | gamma = 0.0001 kernel = linear C = 1 decision_function_shape = ovr (onevsrest) |
